# Multi-Scope Feature Extraction for Intracranial Aneurysm 3D Point Cloud Completion

**DOI:** 10.3390/cells11244107

**Published:** 2022-12-17

**Authors:** Wuwei Ma, Xi Yang, Qiufeng Wang, Kaizhu Huang, Xiaowei Huang

**Affiliations:** 1 School of Advanced Technology, Xi’an Jiaotong-Liverpool University, Suzhou 215123, China; 2 Data Science Research Center, Duke Kunshan University, Kunshan 215316, China; 3 Department of Computer Science, University of Liverpool, Liverpool L69 3BX, UK

**Keywords:** point cloud completion, 3D intracranial aneurysm model repair, multi-scope feature, folding-based decoder, coarse-to-fine

## Abstract

3D point clouds are gradually becoming more widely used in the medical field, however, they are rarely used for 3D representation of intracranial vessels and aneurysms due to the time-consuming data reconstruction. In this paper, we simulate the incomplete intracranial vessels (including aneurysms) in the actual collection from different angles, then propose Multi-Scope Feature Extraction Network (MSENet) for Intracranial Aneurysm 3D Point Cloud Completion. MSENet adopts a multi-scope feature extraction encoder to extract the global features from the incomplete point cloud. This encoder utilizes different scopes to fuse the neighborhood information for each point fully. Then a folding-based decoder is applied to obtain the complete 3D shape. To enable the decoder to intuitively match the original geometric structure, we engage the original points coordinates input to perform residual linking. Finally, we merge and sample the complete but coarse point cloud from the decoder to obtain the final refined complete 3D point cloud shape. We conduct extensive experiments on both 3D intracranial aneurysm datasets and general 3D vision PCN datasets. The results demonstrate the effectiveness of the proposed method on three evaluation metrics compared to baseline: our model increases the F-score to 0.379 (+21.1%)/0.320 (+7.7%), reduces Chamfer Distance score to 0.998 (−33.8%)/0.974 (−6.4%), and reduces the Earth Mover’s Distance to 2.750 (17.8%)/2.858 (−0.8%).

## 1. Introduction

**Figure 1:**
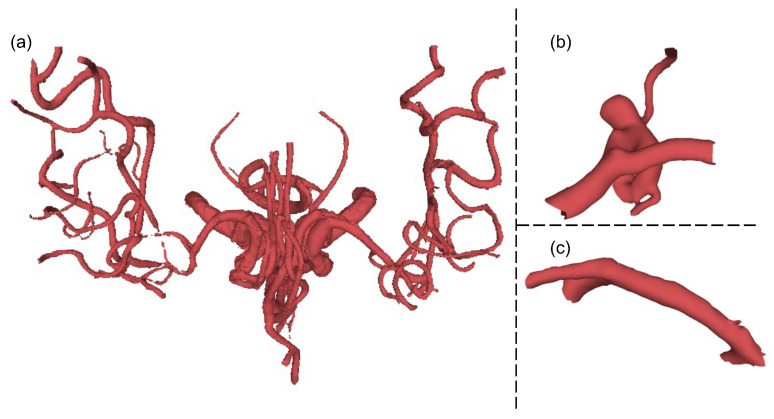
Examples of reconstructing ideal mesh from raw data in IntrA dataset. (**a**) Original raw data built from 2D images. (**b**) Smooth and complete part of aneurysms. (**c**) Smooth and complete part of the healthy vessel.

The intracranial aneurysm can be a life-threatening disease requiring a complicated and costly diagnosis and treatment process. The design of a surgical plan to prevent an aneurysm from rupturing and endangering life is of paramount importance. This kind of surgery requires physicians to operate with pinpoint accuracy since they need clip the neck of aneurysms to prevent rupture. Compared with 2D magnetic resonance angiography (MRA) images, 3D models can provide physicians with more detailed and intuitive information for simulation, diagnosis, and treatment planning. Due to the sensitivity of medical data, there are currently few datasets on intracranial vessels. To our knowledge, the IntrA dataset [1] is the only existing public dataset for intracranial aneurysms neurosurgery simulation, published in 2020, it contains 3D surface models for segmentation and classification in both 3D mesh and point cloud formats. However, the manual preprocessing of raw 3D data is a challenge for experts; the 3D models should be reconstructed to be smooth on their surface (Figure 1), which is a very time-consuming task. For example, the experts spent 50 days to complete this IntrA dataset.

**Figure 2:**
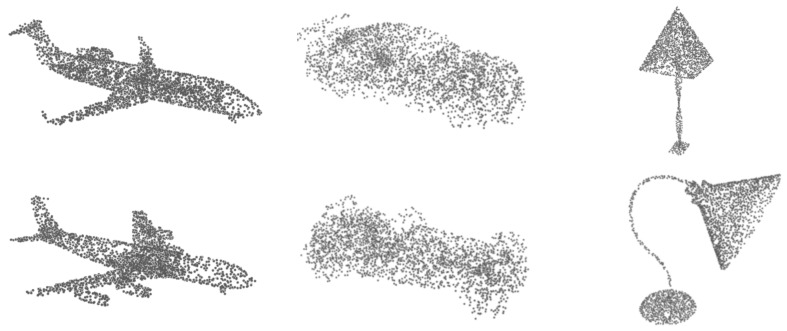
Airplanes, cars, and lamps examples in PCN dataset.

Three-dimensional point clouds have been widely used to represent 3D objects in non-medical fields, because they require less memory than mesh and voxel representations and contain a more comprehensive perspective than multiview images. However, owing to limitations of acquisition devices or occlusions by other objects in the area being imaged [2], 3D point clouds scanned from the real world also suffer from incomplete and uneven shapes, affecting the implementation of downstream tasks, such as segmentation and classification. This problem also occurs in medical fields since CT and MRI are limited by inherent physical limitations [3], noise and ambient light will appear in scanned 2D images, resulting in the rough of 3D models constructed from 2D images. In order to provide more efficient 3D point cloud models for other tasks, point cloud completion has recently attracted significant attention. There are two main challenges in point cloud completion, i.e., feature extraction from the partial shape and completion based on the extracted feature. Based on scholars’ exploration of 3D point clouds, two pioneering works, PointNet [4] and PointNet++ [5], are usually taken as point cloud feature extraction techniques. Specifically, PointNet proposes to extract global features for the three properties of point cloud data format, including unordered points, interaction among points, and invariance under transformations, while PointNet++ partitions the position of each point and aggregates the information of its neighbors to obtain local geometric information. Starting from PointNet, PF-Net [6,7] were proposed based on multi-resolution convolution, which offers richer information for the decoder by aggregating multi-resolution point cloud features. Following feature extraction, the the challenge arises on how to use the decoder to restore the geometric features of the point cloud. Recent attempts [6,8,9,10] have made significant progress in 3D point cloud completion. For example, FoldingNet [11] regards a 3D object as a folding deformation of a 2D grid, which assigns the features extracted by the encoder to each point on the 2D grid, and transforms the 2D grid into a 3D shape through a multilayer perceptron (MLP). Another work AtlasNet [12] splits a 3D object into more 2D planes and obtains the geometry of the 3D object by deforming each plane. However, these works focus mainly on objects with obvious geometric or symmetric structural information, such as airplanes, boats, and lamps (see Figure 2 for illustration).

Despite its importance, there have been few completion tasks in the medical field. In the previous medical-related completion tasks, the objects processed merely miss a small part in their complete shape. For instance, missing teeth completion [13] and skull completion [14]. These instances differ from what is required by our motivation, i.e., generating ideal and smooth 3D models from raw datasets. Different from other common objects, the intracranial vessel and aneurysm fragments are typically asymmetric, with varying geometry and topology. Additionally, the number of data points is limited because of their size. Therefore, the completion model mentioned for ordinary objects may not be suitable for completing intracranial blood vessels and aneurysms.

In this paper, we propose an encoder–decoder-framed deep learning model called the Multi-Scope Feature Extraction Network, which takes one partial intracranial vessel or aneurysm point cloud as its input. we then predicts its complete 3D structure. Concretely, we develop a Multi-Scope feature Extraction encoder to obtain different scope neighborhoods for one point, then extract and fuse the multi-scope features together. For the decoder, we adopt style-based folding using SpareNet [15], but add original partial points coordinates to guide it to recover more structural information from the input. Furthermore, we take the advantage of the pioneer 3D point cloud completion work PCN [2] by utilizing a coarse-to-fine framework to calculate both coarse point cloud and refined point cloud loss between the ground truth with shape completion during the training process.

## 2. Materials and Methods

### 2.1. Data Sources

**Figure 3:**
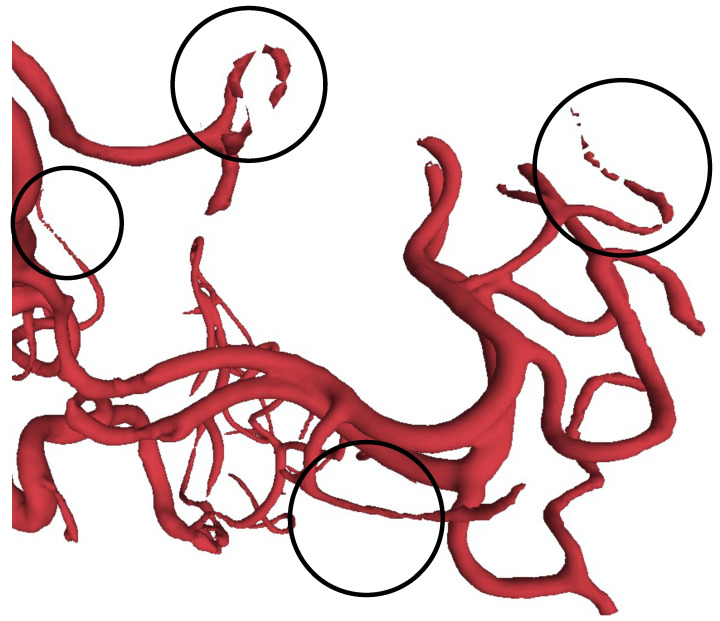
Visualization of incomplete IntrA raw dataset. Incomplete parts are circled.

**Figure 4:**
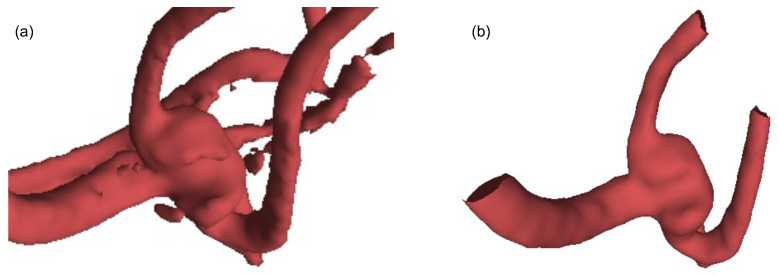
Comparison of IntrA data before and after manually refined: (**a**) The coarse surface before manually refined, (**b**) The smooth surface after manually refined.

In this paper, we adopt the IntrA dataset [1] for the experiments to evaluate the proposed method, which was published in 2020 as a point-based and a mesh-based 3D aneurysm model collection. It contains three data types, including 103 complete models, 1909 generated segments, and 116 annotated segments. The 103 complete brain vessels were reconstructed by scanned 2D MRA images from patients using life sciences software, Amira 2019 (Thermo Fisher Scientific, Waltham, MA, USA), and can take 50 workdays to process [1]. The generated segments are split from the complete models after manually cleaning the data and re-meshing, resulting in 1694 healthy vessels and 215 aneurysms. Each segment has approximately 500 to 1700 points at Geodesic Distance 30. According to IntrA, their dataset is provided to support classification, segmentation, reconstruction. During the IntrA dataset generating process, we found that the authors face the challenge that the restored 3D data model from 2D scanned images were not complete, and could only be restored manually by experts. After carefully studying 103 complete models, we were able to identify incomplete regions as shown in Figure 3 and Figure 4. We can see that even after a long time of reconstruction processing period, the reconstructed 3D models still have noise and their surfaces are not smooth [1].

### 2.2. Experimental Dataset

Some previous works [7,9] set the partial point cloud number equal to the complete point number which transfers the point completion task to a point rearrangement task. This setting is, however, limited by the choice of datasets. The model needs duplicate points or add zero points when the input points are insufficient. The number of output points is also limited to a fixed number. The point cloud completion task can also be seen as a missing part prediction from the existing part [6,16,17] where the final shape isformed by merging the existing partial part and the predicted missing part. These methods need filling the gap between the two parts, where the missing part should shape the same distribution as the input. Since the input shape is incomplete already, its distribution is not guaranteed to be consistent with the complete shape of the ground truth.

Another typical setting that follows PCN [2] is to assume that the input incomplete point cloud and the output complete point cloud are independently distributed. They both represent the same object but are separately sampled from the ground truth. The purpose of this setting is that the incomplete point cloud only provides the model with a clue to predict the complete point cloud, which has structural characteristics similar to the predicted part of the point cloud, but it is not part of the complete point cloud. This setting is more flexible for the number of input and output points set by the network. Since the output point cloud is a complete shape, its distribution is consistent.

Our data setting follows PCN to make a different distribution that is more accurate for intracranial vessel situations. Since we do not have a paired complete model and incomplete model of 103 brain vessels, we choose the IntrA dataset’s 1909 generated segments subset as our dataset. We follow PCN and deal with pure point cloud dataset completion tasks, to generate an extended point cloud completion dataset, called IntrACompletion dataset.

**Figure 5:**
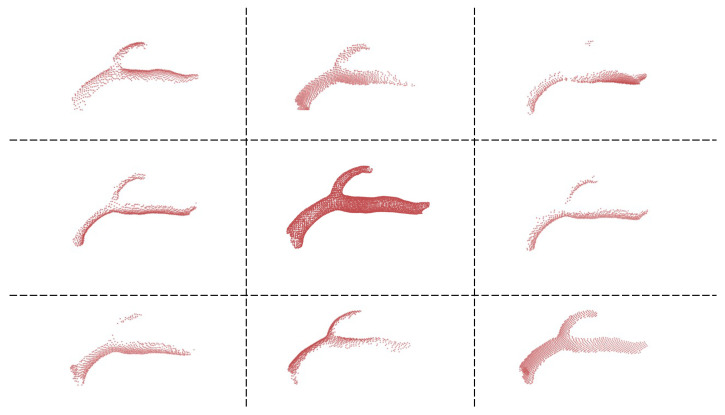
One example of the IntrACompletion dataset. The complete point cloud is shown in the middle, and its 8 different angles partial point clouds are shown to surround it.

**Figure 6:**
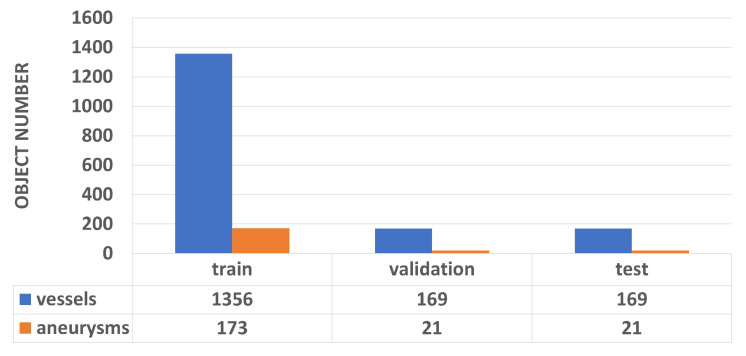
Vessels and aneurysms distribution in the train, validation, and test.

To generate the IntrACompletion dataset from the original IntrA dataset, we take all 1909 segments models, including two categories: 1694 vessels and 215 aneurysms. The complete point clouds are sampled from these 3D meshes uniformly. Similar to PCN, we use back-projected depth images to generate partial point clouds. This operation can separate the distribution of the partial point cloud and its complete shape. More specifically, the partial point clouds are not subsets from their complete point cloud. In addition, we use eight random angles to generate eight different viewpoints of partial point clouds to enrich the dataset (Figure 5). To divide training, validation, and test groups, we use 8:1:1 ratio. That means we have 1356:169:169 for vessels and 173:21:21 for aneurysms (Figure 6) in the following experiments.

### 2.3. Proposed Methods

**Figure 7:**
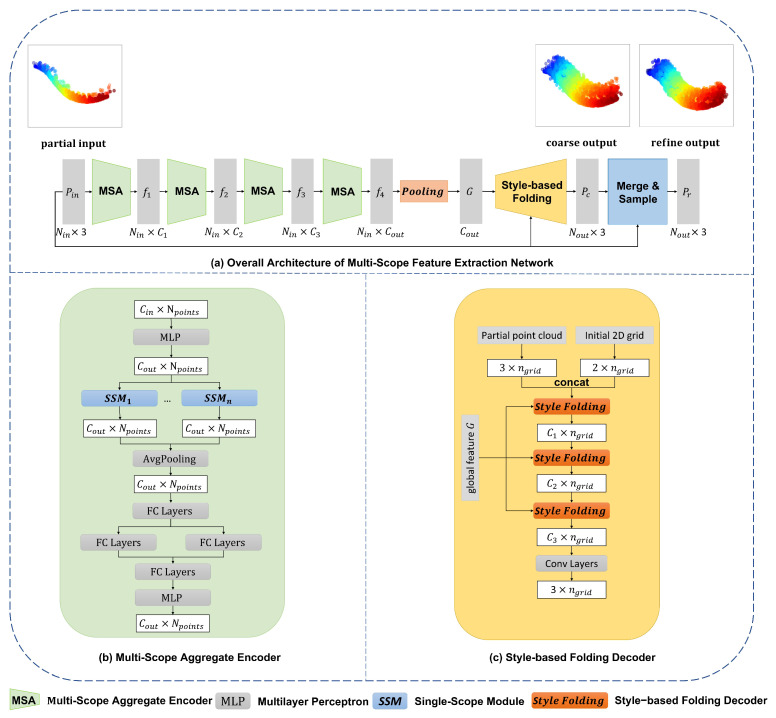
Overall architecture of the proposed Multi-Scope Feature Extraction Network (MSENet). (**a**) Our MSENet contains three modules: a feature extractor built of several MSA modules to get the global features *G* for partial point cloud input, a style-based folding decoder to generate a coarse but complete point cloud, and a merge and sample module to refine the coarse point cloud; (**b**) the detailed structure of the MSA module; and (**c**) the detailed structure of the style-based folding.

In this paper, we proposed a Multi-Scope Feature Extraction Network for intracranial aneurysm vessel point cloud completion on the generated IntrACompletion dataset. This model exploits an encoder–decoder framework and adopts a coarse-to-fine pipeline from PCN [2]. It has a multi-scope aggregate-based encoder to extract global feature *G* from partial input, and a style-based folding decoder predicts the corresponding coarse complete shape. Last, a refinement network is considered to refine the coarse to fine output. The overall architecture is shown in Figure 7a.

#### 2.3.1. Multi-Scope Aggregate Encoder

The characteristic of 3D point clouds is that they are represented by discrete coordinates of points. This representation makes it lighter to store. However, point clouds do not have vertices and surface structures like 3D meshes, therefore, we cannot directly obtain one point’s local features from it. The popular feature extraction method for point clouds is to find a point and its *k* neighbor points with the closest Euclidean Distance, or neighbors within a spherical distance. Aggregating the coordinates and feature information of surrounding points to ensure a point has not only its own features but also local features provided by its neighbors [5,18]. These works usually use a single *k* only in information extraction, resulting in weak performance in real-world datasets with an uneven point distribution. To mitigate this issue, and inspired by VRCNet [19], we propose to apply multi-scope feature extraction. Different from VRCNet, which uses the point self-attention kernel module to select points, we follow [15,20], using EdgeConv [18] to extract features and engage Squeeze-and-Extraction [21] to enhance the features.

**Figure 8:**
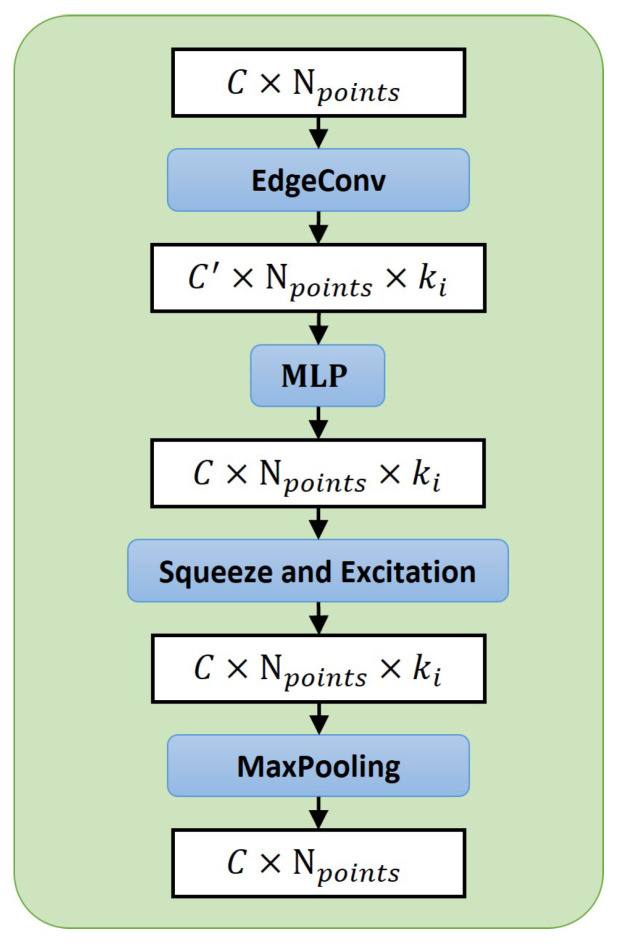
This is the Singe-Scope Module (SSM) which is the main module in our MSA module. Each SSM module is assigned a different *k* value $k_{i}$ in one MSA module.

Our proposed Multi-Scope Aggregate Module (MSA) is shown in Figure 7b. It is combined with several Single-Scope Modules (SSM) (Figure 8). Each SSM module takes an $N\times C_{in}$ unordered partial point cloud as its input, denoted as $P_{in}$. For each point $p_{i}\in P_{in}$, we find its *k*-nearest neighbors (*k*-NN) $p_{i}^{j}\in P_{in}$ and their corresponding features $\left\{e_{i}^{j}\in R^{C_{}}|j=1,2,3,\ldots ,k\right\}$. Then we use EdgeConv [18] to make a directed graph between $p_{i}$ and $p_{i}^{j}$. It combines the local neighborhood information $\left(e_{i}^{j}-e_{i}\right)$ with global information $e_{i}$ on each edge as $\left(e_{i},e_{i}^{j}-e_{i}\right)$. The merged features are sent to a shared MLP to learn each edge’s feature. Accordingly, the learned new features ${e_{i}^{j}}^{\prime }$ of the point $p_{i}$ are assigned as follows:

(1) $${e_{i}^{j}}^{\prime }=\max_{j:(i,j)\in \varepsilon }\mathrm{MLP}\left(e_{i},e_{i}^{j}-e_{i}\right),$$

where ${e_{i}^{j}}^{\prime }\in R^{C_{}},i\in \left\{1,2,\ldots ,N\right\},j\in \left\{1,2,\ldots ,k\right\}$. All new assigned features $e_{i}^{\prime }$ as graph information are squeezed into a channel descriptor through N×k dimensions followed by a squeeze operation in the SE block [21]. We obtain a gating vector *s* with a Sigmoid activation called excitation as follows:

(2) $$s=\sigma (\mathrm{MLP}\left(\frac{1}{N\times k}\sum_{i=1}^{N}\sum_{j=1}^{k}{e_{i}^{j}}^{\prime }\right)).$$

After obtaining new features ${e_{i}^{j}}^{\prime }$ from EdgeConv [18] and the gating vector *s* from the SE block, the MSA layer multiplies the new features by the gating vector. It uses max-pooling to reduce the edges of each graph to find the most representative edge and its features as the final features $N\times C_{out}$ for $P_{in}$ points.

Use of a fixed *k* for the neighborhood may cause unbalanced information. Even if we can rebalance by using multi-resolution processes, such as PFNet [6] and Multiresolution tree networks [7], using one *k* value in different downsampled point sets, the uneven density distribution problem cannot be avoided. To this end, we borrow the R-PSK module provided by VRCNet [19]. The R-PSK module is designed to fuse different scope neighborhood relationships for each point in the point cloud. Our MSA module assigns multiple *k* values for SSM layers. After getting a $C_{in}$ dimensional feature, the MSA module will take one MLP to enhance the feature’s expression ability to $C_{out}$ and send the new higher dimensional feature to multiple SSM layers. Then average-pooling can be applied to merge the features from different scopes. These merged features are used for weights through Softmax and MLP. The weights will guild the feature before SSM modules to realize self-attention.

Our encoder contains four MSA modules which aim to extract different features from low to high-dimension feature space. In the end, the features from the last MSA module will be expanded by both max-pooling and average-pooling to filter important information as global features *G*.

#### 2.3.2. Style-Based Folding Decoder

Folding-based point cloud deformation [2,11,12,22,23,24,25] usually concatenates the global shape, 2D grid, or point cloud coordinates together and learns a mapping between 2D grid and 3D form. This mapping is learned from multiple MLP layers. Since the global shape remains at the first layer, its effect on the overall shape generation is diminished in later layers. To overcome this shortcoming, SpareNet [15] proposes the style-based folding decoder inspired by StyleGAN [26]. It injects the style into each folding internal layer, which is from the global features generated by the encoder. Style-based folding improves the quality of the generated point cloud. Our folding-based decoder borrows SpareNet’s style-based folding decoder that injects style information from global features into MLP layers. Furthermore, we directly add coordinate information of the input point cloud to assist the MLP in capturing more primitive input details.

**Figure 9:**
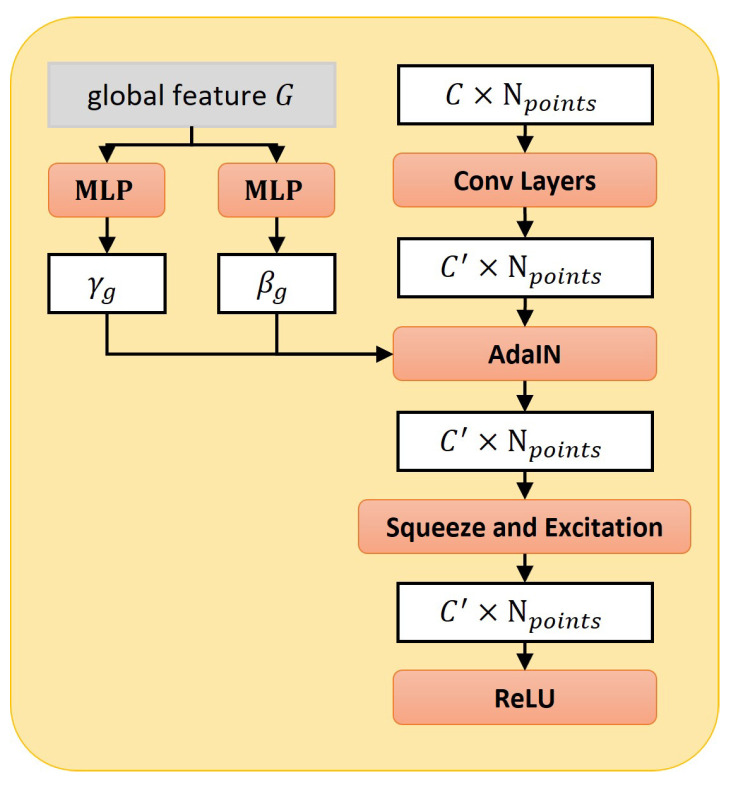
Style-based module with AdaIN (Adaptive Instance Normalization [26]).

In summary, Figure 7c shows that our style-based folding decoder takes three different representations for the object: the global features *G* generated from the MSA encoder, initial 2D grids $P_{n}$, and partial input point cloud $P_{in}$ as inputs. In the style-based module (Figure 9), we learn two modulation parameters $\gamma_{g}$ and $\beta_{g}$ from the global features *G* through MLP, and then utilize an Adaptive Instance Normalization (AdaIN) proposed by StyleGAN [26] to transfer feature $x_{i}$ to new features:

(3) $$x_{i}^{\prime }=AdaIN\left(x_{i}\right),$$

where

(4) $$AdaIN\left(x_{i}\right)=\gamma_{g}\frac{x_{i}-\mu \left(x_{i}\right)}{\sigma \left(x_{i}\right)}+\beta_{g}.$$

In the above, μ and σ denote the mean and variance, respectively. After AdaIN, the feature will be transferred through the SE layer and ReLU to a new feature. As with AtlasNet [12], we assume that each 3D object is combined with *K* patches to *K* decoders (K=32 in our experiments). Each patch is deformed from an initial 2D grid, and each grid is evenly divided into small grids. The vertex coordinates of each small grid are taken as inputs for each decoder. After the style-based folding decoder, each 2D grid learns a mapping to generate a 3D surface. All surfaces are combined directly as a complete coarse shape.

#### 2.3.3. Refinement Module

The 32 surfaces are generated separately. To avoid overlapping between each surface, our model follows MSN [24] by using an expansion penalty (5) and minimum density sampling (MDS) (6) to merge and refine the coarse points from the 32 surfaces as follows:

(5) $$L_{expansion}=\frac{1}{KN}\sum_{1\leq i\leq K}\sum_{\left(u,v\right)\in T_{i}}𝟙\left\{dis\left(u,v\right)\geq \lambda l_{i}\right\}dis\left(u,v\right),$$

where 𝟙 denotes the indicator function sharing the filtering distance that is shorter than $\lambda l_{i}$. Denoting $P_{i}=\left\{p_{j}|1\leq j\leq i\right\}$ as the set of first *i* sampled points, $p_{i}$ is the *i*th sampled point. MSD will return a point that has minimum density as follows:

(6) $$P_{i}=\underset{x\notin P_{i-1}}{\arg\min}\sum_{p_{j}\in P_{i-1}}\exp\left(-{∥x-p_{j}∥}^{2}/\left(2\sigma^{2}\right)\right).$$

In the expansion penalty, every point in each patch will be treated as a vertex set. Moreover, a minimum spanning tree $T_{i}$ [27] is built based on Euclidean distances. Distance between vertex *u* and vertex *v* denotes dis(u,v). Each minimum spanning tree can express the distribution of a patch, and longer distances are penalized until they converge.

#### 2.3.4. Joint Loss Function

Earth Mover’s Distance (EMD) is widely used in the evaluation of point cloud [28], which measures the similarity between the generated point cloud and ground truth (gt) as defined by:

(7) $$L_{EMD}\left(P_{1},P_{2}\right)=\min_{\phi :P_{1}⟶P_{2}}\frac{1}{\left|P_{1}\right|}\sum_{x\in S_{1}}{∥x-\phi \left(x\right)∥}_{2},$$

where ϕ is a bijection. In our experiments, we use EMD loss (7) to train the model. To better guide the model optimization, we set two gt points for the coarse point cloud and refined point cloud, respectively. Both gt points are randomly sampled from the dataset. Finally, we adopt a joint loss method to combine the loss between each point cloud and supervise the network’s learning ability in segments, which is defined by

(8) $$L_{joint}=L_{EMD}\left(P_{coarse},P_{gt\_coarse}\right)+L_{EMD}\left(P_{refine},P_{gt\_refine}\right)+\alpha L_{expansion},$$

where we set α=0.1 in our experiments.

### 2.4. Experimental Setting

Our MSENet is implemented using Pytorch [29] and CUDA. The optimizer is important and it can affect the training result [30], we use the Adam [31] optimizer with $\beta_{1}=0$ and $\beta_{2}=0.9$. The partial input number is 512 and randomly sampled from one of eight angles. The corresponding complete points number is 2048 and randomly sampled from the datasets. We set a batch size of 32, learning rate as 0.0001, and epoch as 150. We train all the models using the joint loss (8) on a single Quadro RTX 8000. To evaluate our proposed model, we use three metrics in the following experiments, including EMD (7), CD (9), and F-score @1%. The CD measure function [28] is calculated by

(9) $$L_{CD}\left(P_{1},P_{2}\right)=\frac{1}{2}\left(\frac{1}{\left|P_{1}\right|}\sum_{x\in P_{1}}\min_{y\in P_{2}}∥x-y∥+\frac{1}{\left|P_{2}\right|}\sum_{y\in P_{2}}\min_{x\in P_{1}}∥x-y∥\right).$$

Comparing the above-mentioned two similarity measure metrics EMD and CD, CD is more computationally efficient while EMD can better capture the shape similarity between two point clouds [32].

### 2.5. Evaluations

We compare our MSENet with other typical methods in this research. AtlasNet [12] generates a point cloud from several patches, FolidingNet [11] reconstructs a point cloud from a 2D grid, and PCN [2] adopts it to generate a complete point cloud. MSN [24] is the baseline of point cloud completion, GRNet [33] proposed a 3DConv gridding method, SpareNet [15] proposes a style-based folding decoder. All the methods are evaluated on two datasets, including our generated IntrACompletion dataset in the medical field and the PCN dataset provided by PCN [2] in the general 3D vision field, and the experimental results are shown in the following section.

## 3. Results

### 3.1. Evaluation on IntrACompletion Dataset

The results are compared in Table 1, Table 2 and Table 3 for the evaluation metrics F-score @1%, CD, and EMD, respectively. We can see that our proposed model offers the best performance on all the metrics for both aneurysm and vessel data point cloud completion, which demonstrates the effectiveness of the proposed method. Compared to point cloud baseline work MSN [24], both our model and MSN exploit 32 surfaces to reconstruct the complete point cloud based on the partial point cloud, but our MSA module takes the multi-scope nearest neighbor range to learn richer features for the decoder. Our MESNet improves the F-score from 0.313 to 0.379, reduces the EMD from 3.345 to 2.750, and 34% lower in CD. Style-based folding then helps each layer of folding obtain global information. Compared to SpareNet [15], which also uses a style-based folding decoder, our model reduces the CD value by 15% and the EMD value by 6%. Our decoder makes this improvement through partial point cooperation. We will discuss the effect of the MSA module and partial point cooperation in Section 3.3 Ablation Study. Comparing the performance over the two types of data for aneurysm and vessel, we can observe that all models have better performance on vessel data because we have more vessel data resulting in model bias to the vessel data.

Note that GRNet [33] was proposed to complete the dense point cloud. It originally uses $64^{3}$ grids to generate and sample a 2048 point coarse cloud from 2048 partial input points, and, finally, generate a 16,384 point dense point cloud. For fair comparison, we use the same ratio to set its network: $32^{3}$ grids to generate and sample a 512 point coarse cloud from 512 partial input points, and finally generate a 2048 point point cloud.

**Figure 10:**
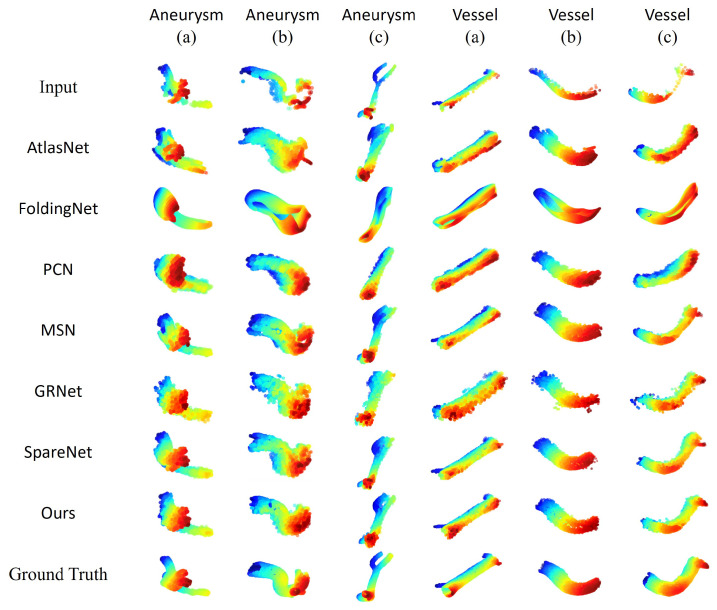
Visual comparison of point cloud completion on the IntrACompletion dataset.

As the purpose of completion is to offer a better 3D model for diagnoses and practices, we make a visualization comparison here. We take six examples from blood vessels and aneurysms for visual comparison. In Figure 10, the first row is the incomplete point cloud input, the following rows are the performance of different models, and the last row is the complete ground truth for comparison. Compared to the folding-based completion models PCN [2], MSN [24], SpareNet [15], we can see that in Aneurysm (a), Vessel (a), and Vessel (c), SpareNet and our model can capture the details of vessel bifurcations, but the others can only recover rough outlines. This is because we apply the style-based folding to inject the global feature in each folding layer. Vessel (b) shows that not every model can reconstruct the tube-like shape. Aneurysm (b) and Aneurysm (c) give us more complex structures.

### 3.2. Evaluation on PCN Dataset

**Figure 11:**
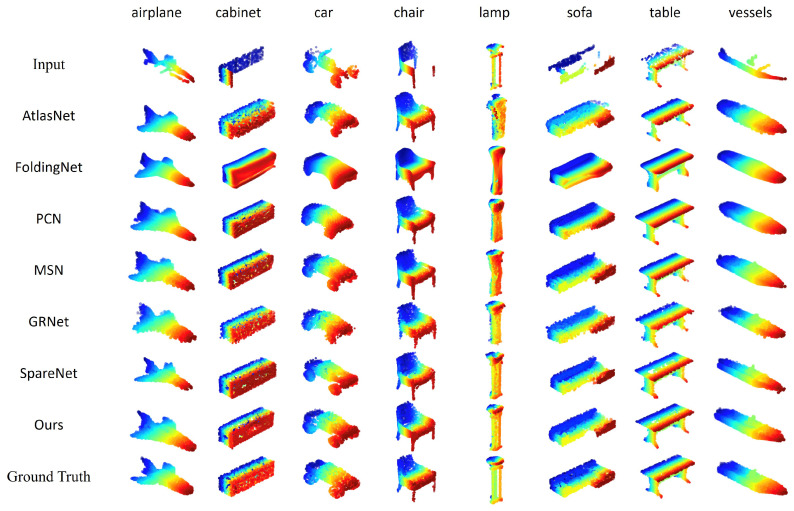
Visual comparison of point cloud completion on the PCN dataset.

To demonstrate the effectiveness of the proposed method in the general point cloud completion in 3D vision, we also conduct the experiments on the PCN dataset [2], which contains airplanes, cabinets, cars, chairs, lamps, sofas, tables, and vessels (boats). For a fair comparison, we use the same train/val/test splits with SpareNet [15] and GRNet [33]. For the overall evaluation, our model is the best in F-score and EMD, as shown in Table 4 and Table 5. For each category, our performance is better in sofa, table, and vessels in Table 6 evaluated by CD, and outperforms 7 out of 8 categories in F-score. The visualization comparison is shown in Figure 11. We can see AtlasNet [12], PCN [2], MSN [24], SpareNet, and our MSENet completing the point cloud using multiple surfaces are able to better represent object surfaces. However, FoldingNet [11] uses a single surface to complete the point cloud, making it hard to separate different parts of objects, such as the wheel section in the third column. In the first column we displayed, due to the missing middle region of the plane the head part of the input point cloud, the full plane head part recovered by MSN and SpareNet is also missing, but our planes head part is complete. In the fifth column, only SpareNet and our model using style-based folding can recover the details of the pole part of the lamp. Compared to SpareNet, our completed point cloud has fewer holes, such as the plane in the first column and the table in the seventh column.

### 3.3. Ablation Study

The proposed method contains two key components, which are MSA module and partial point cooperation. We add them step by step to examine their effectiveness. The ablation studies on IntrACompletion are presented in Table 7. The experimental setting and evaluations are the same as those used in Section 2.4 and Section 2.5. We first set our baseline as using one SSM module assigned a *k* value equal to 5 in our MSA encoder, and remove the partial input for the style-based folding decoder. The EMD score is $3.064\times 10^{3}$, CD score is $1.267\times 10^{3}$, and F-score @1% is 0.354. Compared to the baseline, adding multiple SSM modules in our encoder can reduce $0.091\times 10^{3}$ EMD score, and $0.086\times 10^{3}$ CD score. Partial input can reduce $0.133\times 10^{3}$ EMD score, and $0.149\times 10^{3}$ CD score. The partial input can also increase 0.004 F-score @1% compared to the baseline. With all parts added, the EMD score was reduced $0.314\times 10^{3}$, and $0.296\times 10^{3}$ was reduced in the CD score. Additionally, the two parts increase 0.025 for F-score @1%.

We also performed the ablation study on the PCN dataset as shown in Table 8. Compared to the baseline in the first row, our multiple SSM modules and partial input can reduce the EMD score from $2.890\times 10^{3}$ to $2.858\times 10^{3}$, and enhance the F-score @1% from 0.313 to 0.320. We can note that any addition of MSA module or partial point cooperation can improve the model performance, and MSENet performs better when the two modules are combined.

## 4. Discussion

The development of deep learning in the medical field has been applied to many areas, such as anonymous detection, biological data mining, disease detection, education, etc. [34]. However, some of the open-access data sources listed in the article for disease diagnosis and segmentation only contain hundreds of patient samples. Dataset amount can directly affect the training efficiency, and more samples are helpful. Therefore, how to deal with the original data to make it usable is also worth studying.

Three-dimensional representations are more expressive than two-dimensional data. Furthermore, in medical diagnosis, 3D representations can provide physicians with a clearer and more complete model. Three-dimensional data can help improve the accuracy and efficiency of diagnosis. The full 3D model can also provide clearer objects to practice on in experiments. Since intracranial aneurysm’s are life-threatening, 3D data can provide doctors with better imaging results during diagnosis and aid in developing more accurate surgical plans. The current 3D data are mainly composed of multiple 2D MRA images, and the quality of the synthesis is usually incomplete and noisy, as shown in Figure 3. Such rough data also need be manually refined by experts, which is very time-consuming. We propose a 3D point cloud completion model called Multi-Scope Feature Extraction Network (MSENet) that can complete point clouds of incomplete vessels and aneurysms.

To our knowledge, our model is the first to address the problem of missing data in 3D medical data acquisition. Different from other functional completion problems in the medical field, such as tooth loss [13] or skull completion [14], our model mainly promotes the integrity of the data from the source of data collection and provides more convenient and fast data preprocessing for medical diagnosis and simulation exercises. Lack of data problems are common in the medical field, e.g., in the IntrACompletion dataset which has eight times more vessels than aneurysms as shown in Figure 6. Table 1, Table 2 and Table 3 show that all of the methods show better performance in vessel completion. At present, there is little research considering the differences between different categories when completing point clouds, which we will explore for future research.

## 5. Conclusions

This paper aims to mitigate the incomplete data issue regarding the 3D image reconstruction process for intracranial vessels and aneurysms. We propose a Multi-Scope Feature Extraction Network to complete the partial 3D point cloud. This network engages a multi-scope aggregate module to explore and merge different scopes of neighbors to enhance feature extraction. Then we design a style-based folding decoder to concatenate the original partial points coordinates directly, where the coordinates can offer more existing structure to guide the decoder’s ability. We take the 3D intracranial vessels and aneurysms dataset from IntrA [1] to evaluate our proposed model. We also use a general dataset PCN [2] to confirm this model’s generalizability for other 3D objects. Extensive experimental results demonstrate the effectiveness of the proposed method. Compared to the baseline work MSN [24] on the two datasets, our model increases the F-score to 0.379 (+21.1%)/0.320 (+7.7%), reduces Chamfer Distance score to 0.998 (−33.8%)/0.974 (−6.4%), and reduces the Earth Mover’s Distance to 2.750 (17.8%)/2.858 (−0.8%).

## Figures and Tables

**Table 1:** Point completion results on IntrACompletion in F-score @1% (higher is better).

Methods	Aneurysm	Vessel	Overall
AtlasNet [12]	0.168	0.196	0.193
FoldingNet [11]	0.132	0.161	0.158
PCN [2]	0.140	0.154	0.153
MSN [24]	0.278	0.318	0.313
GRNet [33]	0.224	0.247	0.245
SpareNet [15]	0.322	0.376	0.370
Ours	**0.343**	**0.384**	**0.379**

**Table 2:** Point completion results on IntrACompletion in CD $\times \;10^{3}$ (lower is better).

Methods	Aneurysm	Vessel	Overall
AtlasNet [12]	2.156	2.049	2.061
FoldingNet [11]	5.124	3.781	3.929
PCN [2]	3.972	3.971	3.971
MSN [24]	1.623	1.493	1.508
GRNet [33]	2.068	2.006	2.013
SpareNet [15]	1.453	1.140	1.174
Ours	**1.173**	**0.976**	**0.998**

**Table 3:** Point completion results on IntrACompletion in EMD $\times \;10^{3}$ (lower is better).

Methods	Aneurysm	Vessel	Overall
AtlasNet [12]	4.158	3.983	4.002
FoldingNet [11]	5.401	4.724	4.799
PCN [2]	5.466	5.381	5.391
MSN [24]	3.524	3.323	3.345
GRNet [33]	4.164	4.048	4.061
SpareNet [15]	3.379	2.885	2.940
Ours	**3.000**	**2.719**	**2.750**

**Table 4:** Point completion results on PCN in F-score @1% (higher is better).

Methods	Airplane	Cabinet	Car	Chair	Lamp	Sofa	Table	Vessel	Overall
AtlasNet [12]	0.590	0.096	0.160	0.149	0.211	0.106	0.199	0.263	0.222
FoldingNet [11]	0.509	0.134	0.159	0.161	0.247	0.117	0.229	0.279	0.229
PCN [2]	0.611	0.148	0.200	0.185	0.293	0.128	0.240	0.312	0.265
MSN [24]	**0.670**	0.117	0.202	0.229	0.380	0.148	0.276	0.357	0.297
GRNet [33]	0.554	0.133	0.176	0.197	0.365	0.132	0.233	0.315	0.263
SpareNet [15]	0.664	0.162	0.204	0.240	0.436	0.153	0.275	0.378	0.314
Ours	0.661	**0.164**	**0.206**	**0.249**	**0.439**	**0.167**	**0.297**	**0.380**	**0.320**

**Table 5:** Point completion results on PCN in EMD $\times \;10^{3}$ (lower is better).

Methods	Airplane	Cabinet	Car	Chair	Lamp	Sofa	Table	Vessel	Overall
AtlasNet [12]	1.926	3.809	3.090	3.581	3.920	4.001	3.244	2.879	3.307
FoldingNet [11]	2.090	3.458	3.229	3.711	3.609	3.940	3.188	2.913	3.267
PCN [2]	1.867	**3.261**	**2.720**	3.275	3.268	**3.552**	2.935	2.664	2.943
MSN [24]	**1.704**	3.668	2.844	**3.049**	2.968	3.600	**2.727**	2.486	2.881
GRNet [33]	2.013	3.474	2.954	3.188	2.836	3.705	2.977	2.583	2.966
SpareNet [15]	1.762	3.442	2.913	3.231	**2.691**	3.659	2.937	2.529	2.895
Ours	1.778	3.399	2.889	3.176	2.697	3.600	2.854	**2.472**	**2.858**

**Table 6:** Point completion results on PCN in CD $\times \;10^{3}$ (lower is better).

Methods	Airplane	Cabinet	Car	Chair	Lamp	Sofa	Table	Vessel	Overall
AtlasNet [12]	0.431	1.836	0.977	1.470	1.952	2.014	1.405	0.948	1.379
FoldingNet [11]	0.582	1.746	1.208	1.946	2.203	2.151	1.689	1.182	1.589
PCN [2]	0.408	1.420	**0.775**	1.291	1.234	1.527	1.223	0.816	1.087
MSN [24]	**0.315**	1.691	0.828	**1.153**	1.094	1.607	0.959	0.684	1.041
GRNet [33]	0.441	**1.360**	0.874	1.189	0.994	1.826	1.101	0.691	1.059
SpareNet [15]	0.320	1.374	0.880	1.192	**0.891**	1.501	0.973	0.649	**0.972**
Ours	0.331	1.374	0.874	1.198	0.945	**1.485**	**0.951**	**0.633**	0.974

**Table 7:** Ablation study on partial input in decoder and multi-scope in the encoder using IntrACompletion dataset.

Multi-Scope	Partial Input	EMD $\times \;10^{3}$ (Lower Is Better)	CD $\times \;10^{3}$ (Lower Is Better)	F-Score @1% (Higher Is Better)
		3.064	1.267	0.354
✓		2.973	1.181	0.351
	✓	2.931	1.118	0.358
✓	✓	**2.750**	**0.998**	**0.379**

**Table 8:** Ablation study on partial input in decoder and multi-scope in the encoder using PCN dataset.

Multi-Scope	Partial Input	EMD $\times \;10^{3}$ (Lower Is Better)	CD $\times \;10^{3}$ (Lower Is Better)	F-Score @1% (Higher Is Better)
		2.890	**0.955**	0.313
✓		2.878	0.977	0.312
	✓	2.862	1.011	0.314
✓	✓	**2.858**	0.974	**0.320**

## Data Availability

All datasets used in this paper are public for research usage. IntrA dataset [1] can be found here: [https://github.com/intra3d2019/IntrA, accessed on 6 September 2022], PCN [2] dataset can be found here: [https://github.com/wentaoyuan/pcn, accessed on 9 October 2021].
